# Pan‐genome and multi‐parental framework for high‐resolution trait dissection in melon (*Cucumis melo*)

**DOI:** 10.1111/tpj.16021

**Published:** 2022-11-23

**Authors:** Elad Oren, Asaf Dafna, Galil Tzuri, Ilan Halperin, Tal Isaacson, Meital Elkabetz, Ayala Meir, Uzi Saar, Shachar Ohali, Thuy La, Cinta Romay, Yaakov Tadmor, Arthur A. Schaffer, Edward S. Buckler, Roni Cohen, Joseph Burger, Amit Gur

**Affiliations:** ^1^ Cucurbits Section, Department of Vegetable Sciences Agricultural Research Organization, Newe Ya‘ar Research Center P.O. Box 1021 Ramat Yishay 3009500 Israel; ^2^ The Robert H. Smith Institute of Plant Sciences and Genetics in Agriculture, Faculty of Agriculture The Hebrew University of Jerusalem Rehovot Israel; ^3^ Institute for Genomic Diversity, Cornell University Ithaca New York 14853 USA; ^4^ Department of Vegetable Sciences Institute of Plant Sciences, Agricultural Research Organization, The Volcani Center P.O. Box 15159 Rishon LeZiyyon 7507101 Israel; ^5^ United States Department of Agriculture‐Agricultural Research Service Robert W. Holley Center for Agriculture and Health Ithaca New York 14853 USA

**Keywords:** pan‐genome, *Cucumis melo*, structural variation, SNP, genetic mapping, half‐diallel, crop breeding, fruit‐quality, disease resistance

## Abstract

Linking genotype with phenotype is a fundamental goal in biology and requires robust data for both. Recent advances in plant‐genome sequencing have expedited comparisons among multiple‐related individuals. The abundance of structural genomic within‐species variation that has been discovered indicates that a single reference genome cannot represent the complete sequence diversity of a species, leading to the expansion of the pan‐genome concept. For high‐resolution forward genetics, this unprecedented access to genomic variation should be paralleled and integrated with phenotypic characterization of genetic diversity. We developed a multi‐parental framework for trait dissection in melon (*Cucumis melo*), leveraging a novel pan‐genome constructed for this highly variable cucurbit crop. A core subset of 25 diverse founders (*MelonCore25*), consisting of 24 accessions from the two widely cultivated subspecies of *C. melo*, encompassing 12 horticultural groups, and 1 feral accession was sequenced using a combination of short‐ and long‐read technologies, and their genomes were assembled *de novo*. The construction of this melon pan‐genome exposed substantial variation in genome size and structure, including detection of ~300 000 structural variants and ~9 million SNPs. A half‐diallel derived set of 300 F_2_ populations, representing all possible *MelonCore25* parental combinations, was constructed as a framework for trait dissection through integration with the pan‐genome. We demonstrate the potential of this unified framework for genetic analysis of various melon traits, including rind color intensity and pattern, fruit sugar content, and resistance to fungal diseases. We anticipate that utilization of this integrated resource will enhance genetic dissection of important traits and accelerate melon breeding.

## Significance Statement

Pan‐genomes aim to address the abundance of genome structural variation within species for improved genomic analyses. A new pan‐genome, constructed from *de novo* genome assemblies of 25 diverse melon (*Cucumis melo* L.) accessions, is integrated with a half‐diallel derived set of 300 F_2_ populations representing all possible parental combinations. The potential of this unified multi‐parental trait‐dissection framework for melon genetics and breeding is presented.

## INTRODUCTION

The ability to investigate gene functions in organisms has been revolutionized in recent years by the extensive implementation of genome editing in genetic research, through CRISPR‐Cas9 technology (Doudna & Charpentier, 2014). Nevertheless, genetic dissection of simple or complex traits in crop plants is still fundamentally dependent on forward‐genetics approaches, including the characterization of natural variation followed by genetic mapping of major Mendelian genes and quantitative trait loci (QTL). Genetic dissection of traits and QTL mapping have been accelerated by the rapid evolution of sequencing and genotyping technologies during the past two decades (Purugganan & Jackson, 2021). Parallel‐sequencing‐based genotyping approaches supported by multiplexing protocols (Baird et al., 2008; Elshire et al., 2011) enable high‐throughput and cost‐effective dense genome‐wide genotyping of populations. This technology‐driven advancement facilitates efficient genotypic screening of large populations, and is shifting the bottleneck in mapping studies from genotyping to the phenotypic characterization of genetic diversity. Therefore, the development and availability of effective diverse germplasm and segregating populations for genetic mapping within species is an important factor.

Access to plant whole‐genome assemblies in the early 2000s opened new avenues in crop genetics, and further straightened the path from traits to QTLs and genes (Morrell et al., 2011). The ability to use reference genomes for physical mapping and annotation of QTL intervals has been instrumental for moving from QTLs to causative genes. An important hidden layer in the genetic variation was only recently exposed through the assembly and comparisons of multiple within‐species reference genomes. The discovery of extensive intraspecific structural variation (SV) led to the realization that a single reference genome does not represent the diversity within a species, and led to the expansion of the pan‐genome concept (Bayer et al., 2020; Coletta et al., 2021).

Melons (*Cucumis melo* L., *Cucurbitaceae*) are among the most widely consumed fleshy fruits for fresh consumption worldwide (http://faostat3.fao.org/). Melons have been bred and are cultivated in nearly all of the warmer regions of the world, leading to the evolution of extensive diversity in phenotypic traits, especially in fruit traits such as size, shape, exocarp (rind) and mesocarp (flesh) color, sugar content, acidity, texture and aroma (Burger et al., 2006). This wide diversity is a source for further breeding and ongoing genetic research aimed at mapping QTLs and identifying genes affecting key horticultural and consumer‐preference traits. Numerous genetic studies using diverse collections or segregating populations have focused on quality traits in melon, including fruit size and shape (Martínez‐Martínez et al., 2022; Monforte et al., 2014; Oren et al., 2020), flesh color (Cuevas et al., 2009; Tzuri et al., 2015), rind color (Feder et al., 2015; Oren et al., 2019), netting and ‘sutures’ (Diaz et al., 2011; Oren et al., 2020; Zhao et al., 2019), sweetness and aroma (Argyris et al., 2017; Harel‐Beja et al., 2010), acidity (Cohen et al., 2014) and ripening behavior (Oren et al., 2022; Pereira et al., 2020; Ríos et al., 2017). Multiple analyses have also been performed for identification and genetic characterization of resistance to pathogens (Burger et al., 2003; Cohen et al., 2022; Joobeur et al., 2004).

Since 2012, mapping studies in melon have benefited from the availability of a reference genome (Garcia‐Mas et al., 2012) enabling physical mapping and annotations of QTL intervals. Resequencing of hundreds of melon accessions (Liu, Gao, et al., 2020; Zhao et al., 2019) in recent years has further advanced the genomic analysis of candidate and causative genes. Five additional genome assemblies were published for *Cucumis melo* during the last 4 years. Three of these were of susp. *Melo*: ‘Payzawat’ from the Inodorus Group (Zhang et al., 2019), ‘Harukei‐3’ from the Reticulatus Group (Yano et al., 2020), and ‘Charmono’ from the Cantalupensis Group (Pichot et al., 2022). The other two were of susp. *Agrestis*: HS (Yang et al., 2020) and IVF77 (Ling et al., 2021). These assemblies provide additional references and important information regarding variation in *C. melo* genome structure, but a wider pan‐genomic view is still lacking for this species.

In the current study, we describe an integrated multi‐parental framework that we developed for genetic dissection of traits in melon. This framework is based on a representative core subset of 25 diverse melon inbred accessions that was used to construct a wide set of 300 segregating bi‐parental populations derived from a 25 × 25 half‐diallele. A novel pan‐genome that we built from the sequencing and *de novo* assemblies of the 25 genomes is integrated with the multi‐parental germplasm resource to create a unified trait dissection framework. We provide here examples for the effectiveness and potential of this resource for genetic analysis of various phenotypic traits in melon.

## RESULTS AND DISCUSSION

### Selection of 25 founders from the GWAS180 diversity panel and development of the HDA25 and HDA25F_2_ populations

**Figure 1:**
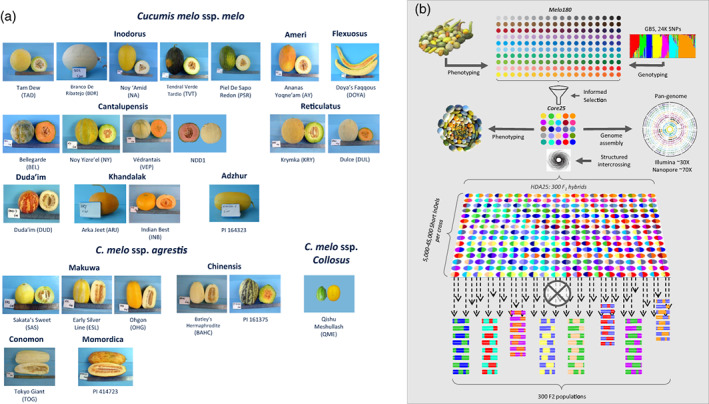
The *MelonCore25* set and mapping platform development scheme. (a) Mature fruits of the 25 founders, ordered by subspecies and horticultural (cultivar‐) groups. (b) Schematic diagram for the components and development process of the multi‐parental framework.

Our primary resource in the current project is the diverse melon collection maintained at Newe Ya‘ar Research Center. This collection contains many accessions drawn from the two widely cultivated subspecies of *C. melo* (ssp. *melo* and ssp. *agrestis*) and includes representatives of 12 horticultural groups. This collection has been characterized for a wide range of phenotypic traits, and genotyped with ~24 000 genome‐wide GBS‐based SNP markers (Gur et al., 2017). Integration of the combined subspecific/horticultural‐group classification with the phenotypic and genotypic data facilitated the informed selection of a balanced core subset composed of 25 founders that represent the overall diversity of *C. melo* (hereafter *MelonCore25*; Figure 1a,b; Table 1; Gur et al., 2017). As such, the core set includes representatives of the two cultivated sub‐species and the different horticultural groups in melon as well as the broad phenotypic spectrum available for key traits, as previously described (Gur et al., 2017). The core set includes 17 accessions of ssp. *melo* (according to cultivar‐group: 5 Inodorus, 4 Cantalupensis, 2 Reticulatus, 2 Khandalak, 1 Duda'im, 1 Flexuosus, 1 Ameri and 1 thought to be Adzhur), 7 accessions of ssp. *agrestis* (according to cultivar‐group: 2 Chinensis, 1 Conomon, 3 Makuwa and 1 Momordica), and 1 feral accession, ssp. *collosus*, collected in central Israel.

**Table 1:** List and taxonomic affiliation of the 25 founders of *MelonCore25* and their genome assembly statistics

Line short name	Variety name	Sub‐species	Varietytype	Assembly size (Mb)	Total number of contigs	Total number of scaffolds	Largest contig (Mb)	Contigs size N50 (Mb)	Contigs size N90 (Mb)	Assembly Complete BUSCOs (% of 2325 genes)	LAI	Number of annotated genes
ARJ	Arka Jeet	*melo*	Khandalak	364.95	525	327	14.29	4.90	1.48	97.94%	13.22	36 773
AY	Ananas Yoqne'am	*melo*	Ameri	367.46	280	176	22.86	8.18	2.25	97.85%	13.28	37 183
BAHC	Bately's Hermaphrodite	*agrestis*	Chinensis	361.94	247	170	22.73	11.24	3.19	97.94%	12.19	36 981
BDR	Branco De Ribatejo	*melo*	Inodorus	365.98	462	346	24.52	7.92	2.85	97.85%	13.20	37 136
BEL	Bellegarde	*melo*	Cantalupensis	373.83	226	142	23.52	8.03	3.09	97.76%	14.15	37 193
DOYA	Doya's Faqqous	*melo*	Flexuosus	366.72	473	277	21.03	5.45	1.72	97.72%	13.05	36 513
DUD	Duda'im 3	*melo*	Duda'im	362.85	357	214	16.18	5.78	1.78	97.89%	11.90	36 602
DUL	Dulce	*melo*	Reticulatus	365.53	124	63	22.65	10.68	3.17	97.63%	11.87	36 175
ESL	Early Silver Line	*agrestis*	Makuwa	358.58	560	163	8.45	1.94	0.48	97.94%	11.11	36 345
INB	Indian Best	*melo*	Khandalak	363.58	231	117	24.99	6.04	2.16	97.89%	12.88	36 626
KRY	Krimka	*melo*	Reticulatus	368.44	441	295	34.51	9.72	1.58	97.76%	13.23	37 158
NA	Noy ‘Amid	*melo*	Inodorus	367.29	239	155	27.05	12.63	3.11	97.85%	13.98	37 259
NDD1	NDD1	*melo*	Cantalupensis	365.12	282	208	40.92	10.69	2.99	97.85%	14.30	37 122
NY	Noy Yizre'el	*melo*	Cantalupensis	365.69	221	148	23.95	12.55	2.96	97.85%	13.48	36 919
OHG	Ohgon	*agrestis*	Makuwa	360.67	174	113	23.11	13.81	3.53	97.94%	12.36	36 883
PI161375	PI161375	*agrestis*	Chinensis	360.06	458	242	15.99	4.29	0.99	97.85%	11.65	36 593
PI164323	PI164323	*melo*	Adzhur	367.92	1029	750	22.87	5.69	0.81	98.02%	12.58	36 394
PI414723	PI414723	*agrestis*	Momordica	363.40	230	157	22.82	11.89	3.30	98.06%	12.80	36 458
PSR	Piel De Sapo Redon	*melo*	Inodorus	368.71	258	165	26.60	9.26	2.91	97.89%	13.40	37 232
QME	Qishu Meshullash	*C. collosus*	feral	363.55	269	184	32.87	15.49	2.90	98.02%	13.10	36 578
SAS	Sakata's Sweet	*agrestis*	Makuwa	361.03	432	309	20.76	6.75	2.27	97.94%	11.74	36 725
TAD	Tam Dew	*melo*	Inodorus	364.88	173	98	23.60	12.13	3.14	97.94%	13.33	37 120
TOG	Tokyo Giant	*agrestis*	Conomon	361.17	273	161	21.63	9.11	1.73	97.94%	12.26	36 802
TVT	Tendral Verde Tardio	*melo*	Inodorus	364.82	245	142	42.25	7.38	2.44	97.89%	13.45	36 970
VEP	Védrantais	*melo*	Cantalupensis	365.38	528	392	22.55	6.88	1.60	97.81%	13.33	36 984
DHL92v4.0				**357.74**	**1192**	**1192**	**5.66**	**0.69**	**0.19**	**97.55%**	**12.99**	**28 299**
Harukei‐3		*melo*	Reticulatus	**372.24**	**138**	**138**	**23.54**	**8.25**	**1.95**	**97.76%**	**13.51**	**37 254**

LAI, LTR Assembly Index.

bold values highlight the two reference genomes used for comparison.

To fully exploit the genetic diversity of this multi‐parental resource, we developed a half‐diallel population by intercrossing the 25 founders in all possible combinations (*HDA25*; Dafna et al., 2021). This crossing scheme was further advanced by self‐pollinations of all the F_1_s to produce 300 distinct F_2_ populations, now available for genetic dissection of melon traits (hereafter *HDA25F*_*2*_ *s*; Figure 1b).

### Sequencing, *de novo* assembly of 25 chromosome‐scale genomes and characterization of structural genomic variation

**Figure 2:**
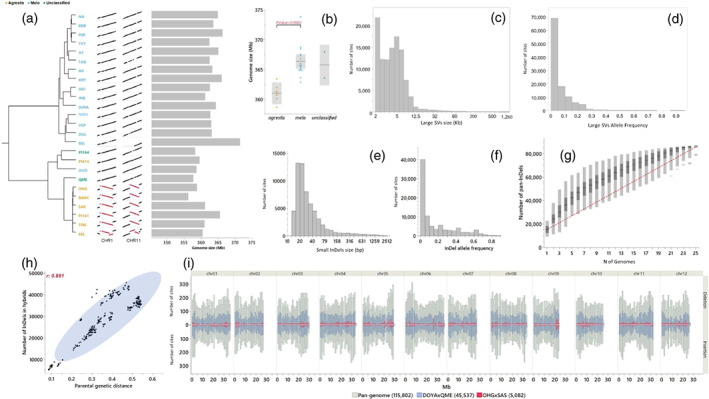
Melon pan‐genome and distribution of structural variations (SVs) across *HDA25*. (a) Phylogenetic tree of the 25 founder lines presented with variation in genome size and examples of large inter‐chromosomal inversions on chromosomes 1 and 11 in ssp. *agrestis*. (b) The genomes of the ssp. *agrestis* accessions are significantly smaller than those of the ssp. *melo* accessions. (c) Size distribution of 108 011 large SVs (> 2 kb). The *X*‐axis is presented in log scale. (d) Distribution of allele frequency of 108 011 large SVs (> 2 kb). (e) Size distribution of 86 669 short (< 2 kb) bi‐allelic InDels across *MelonCore25*. The *X‐*axis is presented in log scale. (f) Distribution of allele frequency across 86 669 bi‐allelic sites (< 2 kb). (g) Pan InDels analysis. Horizontal black lines are the mean number of pan InDels under the corresponding number of genomes. Dark boxes are the 50th percentile and light boxes are the 99th percentile. (h) Correlation between parental distance (calculated using 24 000 genome‐wide GBS SNPs) and number of polymorphic short InDels across the 300 *HDA25* crosses. (i) Number of polymorphic short InDels by chromosomes and bins, in the pan‐genome and two crosses. In parentheses is the total number of short Indels for each group.

The 25 genomes comprising *MelonCore25* were sequenced by Oxford Nanopore Technology (ONT) to an average depth of 70 × (53–134 ×), generating a total sequence of 642.4 Gb with a median read length of 10 kb (4.5–24 kb) and a median read quality of 10.0 (9.2–10.6; Table S1). ONT reads were assembled into contigs and then polished using Illumina short‐reads from the previous whole‐genome resequencing of these accessions (average depth 30 ×; Dafna et al., 2021). The mean N50 of contig size across the 25 genomes was 8.4 Mb and the average number of contigs per genome was 349.5 (median 273; Table 1). The contigs were scaffolded into pseudomolecules through a reference‐guided approach using ‘Harukei‐3’ (Yano et al., 2020) as the reference. Assembly statistics across the *MelonCore25* exposed variation in genome size ranging from 358 Mb (ESL) to 374 Mb (BEL), with a mean of 365 Mb (Table 1). These assembly statistics are comparable to the two published high‐quality melon reference genomes, DHL92 v4.0 (Castanera et al., 2020) and ‘Harukei‐3’ (Yano et al., 2020). Additional quality benchmarks for our assemblies are the high gene‐space integrity and completeness, based on BUSCO scores that ranged from 97.5% to 98.1% complete genes, and the completeness of transposable elements based on the LTR Assembly Index (LAI) with an average of 12.9 (Table 1). The 25 genomes were annotated for gene models, and using stringent parameters (described in Experimental Procedures section) we found variation ranging from 36 175 to 37 259 genes per genome that was not correlated with genome size (Table 1). Interestingly, genome size variation was correlated with the sub‐specific division such that the genomes of ssp. *agrestis* accessions were significantly smaller (mean 361 Mb) than those of the ssp. *melo* accessions (mean 366.3 Mb; Figure 2a,b). Alignment of the 25 genomes and analysis of SV revealed extensive polymorphism in all types of SVs (InDels, inversions and translocations). Large intra‐chromosomal inversions spanning across 1.6 and 3.2 Mb that differentiated between most *agrestis* and *melo* lines were found in chromosomes 1 and 11, respectively (Figure 2a). The independent discovery of these inversions across multiple accessions is further evidence for the reliability of our assemblies. We identified 108 000 large SVs (InDels and translocations) ranging from 2 to 1300 kb (Figure 2c; Table S2). Sixty‐three percent of these, 68 500 SVs, were rare and 39 500 SVs were present in two or more accessions (Figure 2d). We then focused on characterization of short InDels that are useful as simple polymerase chain reaction (PCR)‐based genetic markers in mapping studies. We identified 190 543 short InDel alleles in a size range of 10–2000 bp (Figure 2e; Table S3). A significant proportion of these InDels represent multi‐allelic polymorphisms and converged into 115 802 sites. Allele frequency analysis across the 86 669 bi‐allelic sites showed a similar distribution pattern as in the large SVs, such that almost half of these InDels were rare (Figure 2f). To confirm that *MelonCore25* comprehensively represents the genomic diversity in melon, a pan‐genome analysis for the 86 669 bi‐allelic InDels was performed by plotting the number of cumulative InDels against the number of genomes. The results showed that 22 accessions were sufficient to capture 98% of the SV (Figure 2g). The short InDel profiles of the parents were then projected on the 300 *HDA25* crosses to obtain a detailed description of the polymorphisms in each cross (Table S3). The number of polymorphic sites per cross range from 5082 to 45 537 and, as expected, was strongly correlated with parental genetic distance previously calculated based on 24 000 GBS SNPs (Figure 2h). Short‐InDels coverage across the 12 melon chromosomes was relatively uniform in the *MelonCore25* pan‐genome and specific crosses, regardless of the mating distance (Figure 2i). Even in the cross between the two closely related *agrestis* accessions, OHG and SAS, we identified more than 400 short InDels per chromosome. A random set of 22 short InDels from five of the chromosomes was validated by PCR (Figure S2; Table S4).

Genome sequences are available to date for 18 species of *Cucurbitaceae*. These cucurbit genomes exhibit much variation in size and organization (Ma et al., 2022). Information on intra‐specific variation in genome size within this family is, however, still relatively limited. Genomes of 11 *Cucumis sativus* L. (cucumber) accessions were recently sequenced and compared, exposing a difference of 26 Mb (11%) between the largest and smallest genomes, as well as 56 214 SVs (Li et al., 2022). We observed size variation of 16 Mb (4.5%) between the largest and smallest genomes across *MelonCore25*. The difference in genome size was significant between the *melo* and *agrestis* sub‐species (Figure 2b), and implies the possible evolutionary significance of within‐species genome size variation (Biémont, 2008). The variation in genome size and the consistently differential, large chromosome inversions (Figure 2a) imply that these two subspecies have evolved separately under cultivation, with little or no interaction. Parallel evolution under domestication for fruit‐quality traits has also been described for the two cultivated subspecies of another cucurbit, *Cucurbita pepo* L. (squash/pumpkin; Paris et al., 2012).

### Workflow for utilization of the multi‐parental framework

Our integrated framework relies on the connection of the structured multi‐parental set of populations (*HDA25F*_*2*_ *s*) with phenotypic data, whole‐genome assemblies and the derived variome of the 25 parents (Tables S2 and S3). The proposed utilization workflow is described in Figure S1. Briefly, a trait of interest should first be scanned across *MelonCore25* by either using the database for the trait, or by direct phenotypic analysis. Following reliable phenotyping, we found that projecting the phenotypic data on the 2D genetic PCA plot of *MelonCore25* (coordinates available in Table S1) is a useful way to integrate genotype and phenotype for informed selection of parents differing in the trait of interest and reflecting varying parental genetic distances. Following the selection of parents, an optimal F_2_ population can be obtained from the *HDA25F*_*2*_ *s* set for characterization of the mode of inheritance and genetic mapping of the trait. Depending on the genetic architecture and complexity of the trait, the relevant mapping strategy can be applied and, accordingly, the population size and generation can be adjusted. Parental genome assemblies of the selected population are available as reference for bulk‐sequencing analyses (BSA‐Seq, QTL‐Seq) and for marker development for further fine‐mapping. Detailed annotation of simple or QTL intervals can also benefit from referring to the specific parental genomes. After the initial whole‐genome mapping, the *HDA25F*_*2*_ *s* set can be a valuable resource for selection of additional relevant crosses for QTL validation or allelism testing. Genomes of the 25 founders can then be used for mining allelic variation at candidate genes.

### Examples for implementation

#### Genetics of pigment intensity and mottled rind

**Figure 3:**
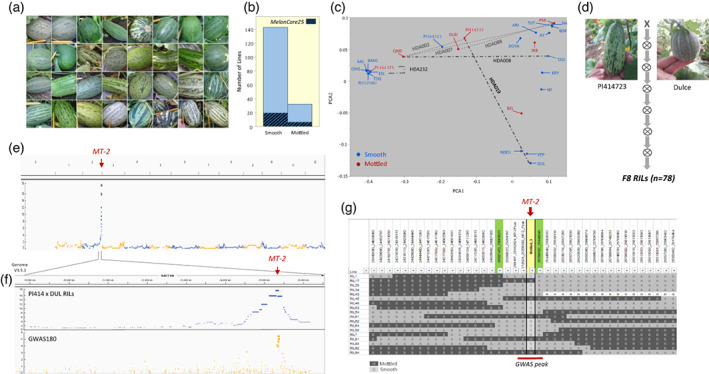
Genetic analysis and mapping of mottled rind. (a) Representative mottled rind fruits from the *GWAS180* collection. (b) Parallel frequencies of mottled‐ and non‐mottled rind accessions in the *GWAS180* collection and the *MelonCore25* set. (c) Projection of rind pattern (mottled or non‐mottled) on the genetic PCA plot of the *MelonCore25* set. Dashed lines indicate crosses (and derived segregating populations) used for genetic analysis of the mottled‐rind trait. (d) PI414723 and DUL, the parents of the RILs population used for mapping the mottled‐rind trait. (e) Linkage mapping results of the *MT‐2* locus to chromosome 2. (f) Zoom in on chromosome 2 mapping in the RILs of the cross PI414723 × DUL and comparison with a Manhattan plot derived from genome‐wide association (GWA) analysis of the *GWAS180* collection. (g) Substitution‐mapping validation for the *MT‐2* locus using recombinants from the RILs of the cross PI414723 × DUL. Green cells represent flanking markers for the interval. The yellow cell is the rind phenotype column. A = DUL allele, B = PI414723 allele. The genome‐wide association study (GWAS) peak interval is represented by the red horizontal line.

Mottled rind is a common characteristic in melon and is expressed in different patterns across our *GWAS180* collection (Figure 3a). As with other traits, *MelonCore25* captures this diversity with parallel proportions of mottled and non‐mottled rind, as in the wider *GWAS180* collection (Figure 3b). To present a comprehensive description of its genetic architecture, the rind‐mottling trait was projected on the genetic PCA plot, and several mottled and non‐mottled accessions were selected for focused genetic analyses (Figure 3c). First, mottled rind was mapped, using the cross between ‘Dulce’ (DUL, ssp*. melo*, Reticulatus Group), which has a uniformly dark rind, and the mottled‐rind PI414723 (PI414, ssp*. agrestis*, Momordica Group), using a previously described RILs population (Figure 3d; Galpaz et al., 2018). Whole‐genome linkage analysis resulted in the mapping of a single major trait locus to ~400 kb interval on chromosome 2 (Figure 3e). The association of this locus with mottled rind was validated through independent genome‐wide association (GWA) analysis of this trait on our large collection (Figure 3f; *GWAS180*, *n* = 177). The integration of linkage and association analyses allowed us to narrow the mapping to a common genomic interval of less than 200 kb, which was further confirmed through substitution mapping using a detailed analysis of recombinants obtained from the PI414 × DUL RILs (Figure 3g).

**Figure 4:**
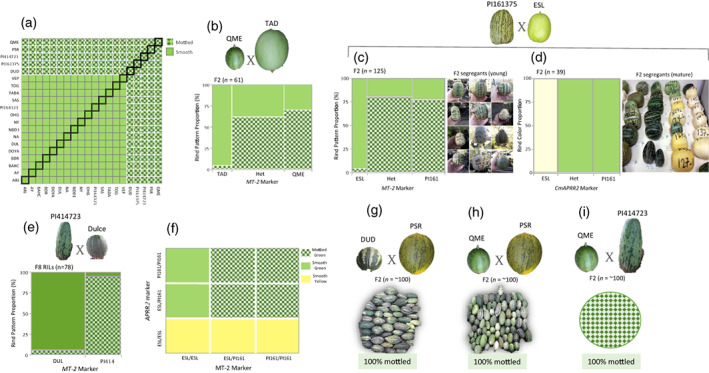
Multi‐parental characterization of the mottled rind trait. (a) Inheritance of rind pattern across the *HDA20* set (190 half‐diallel F_1_s and their 20 parents). Each cell represents the combination of parents from the horizontal and vertical axes. Squares arranged in the diagonal represent the phenotypes of the parents. (b) Contingency analysis for the segregation of rind pattern against the *MT‐2* marker in the F_2_ of the cross QME × TAD. Het = Heterozygote. (c) Contingency analysis for the segregation of rind pattern against the *MT‐2* marker in the F_2_ of the cross PI161375 × ESL. Het = heterozygote. Examples for young fruit segregants (mottled and non‐mottled) are displayed. (d) Contingency analysis for the segregation of rind color against the *APRR2* marker in the F_2_ of the cross PI161375 × ESL. Het = heterozygote. Examples for mature fruit segregants (light and dark rind) are displayed. (e) Contingency analysis for the segregation of rind pattern against the *MT‐2* marker in the RILs from the cross PI414723 × DUL. (f) Schematic pivot table that presents the co‐segregation of the *APRR2* and *MT‐2* markers against rind color and pattern in the F_2_ of the cross PI161375 × ESL. (g) Allelism test for the mottled‐rind trait in the F_2_ of the cross DUD × PSR. (h) Allelism test for the mottled rind trait in the F_2_ of the cross QME × PSR. (i) Allelism test for the mottled rind trait in the F_2_ of the cross QME × PI414723.

To expand the analysis of the mode of inheritance of the mottled‐rind trait, rind pattern of young fruits was examined across the *HDA20* population (subset of 20 founders from *MelonCore25*, and their 190 half‐diallel F_1_s). All crosses with a mottled parent resulted in mottled F_1_s, and non‐mottled F_1_s were obtained only by the combination of two non‐mottled parents (Figure 4a). The results from F_2_ populations were also consistent with a single dominant gene for mottled rind. Subsequently, two additional segregating populations from crosses between mottled and non‐mottled parents were examined. One was an F_2_ population from the cross between the mottled ssp. *collosus* accession QME and the non‐mottled, ssp. *melo*, Inodorus Group TAD (Figure 4b). The other was an F_2_ population from the cross between two closely related ssp. *agrestis* accessions, the mottled PI161375 (Chinensis Group) and the uniformly light, non‐mottled ESL (Makuwa Group; Figure 4c). In both populations, rind mottling was phenotyped on young fruits (10–15 days post‐anthesis). A significant association was observed between the markers that we developed at the *MT‐2* locus and mottled‐rind segregation (Figure 4b,c). Interestingly, segregation and association patterns in these populations were different when compared with the PI414 × DUL RILs population (Figure 4e). In both crosses, groups homozygous to the non‐mottled allele at the *MT‐2* marker were perfectly associated with non‐mottled rind, but the heterozygotes and homozygotes for mottled‐rind allele included also a significant proportion of non‐mottled rind segregants (Figure 4b,c,e). Such a pattern suggests that epistasis with another locus is affecting the expression of this trait. Recently, Shen et al. (2021) proposed that *CmAPRR2* is epistatic over *MT‐2* in controlling mottled rind phenotype in a cross between the mottled *agrestis* inbred (‘Songwhan Charmi’) and a non‐mottled *ssp. melo* ‘Mi Gua’. The PI161375 × ESL population displayed clear segregation to light and dark rind color, and was therefore also used to test the association between *CmAPRR2* gene and rind pigment intensity in mature fruits. In a previous study, we proposed that the light rind of ESL is a result of a single‐base insertion in exon9 of the *CmAPRR2* gene, causing a frameshift that leads to a major modification in the predicted protein sequence (Oren et al., 2019). PI161375 carries the normal (‘dark’) allele of this gene and, indeed, complete co‐segregation was observed between the SNP in *CmAPRR2* and rind color, supporting the proposed exon9 polymorphism as causative (Figure 4d). When combined, the genotypic data from the *CmAPRR2* and the *MT‐2* markers in the ESL × PI161375 population confirmed that the segregation of the two genes can perfectly explain the mottled and non‐mottled rind segregation (Figure 4f), with the light allele of *CmAPRR2* epistatic over *MT‐2*.

To further validate the assumed allelism among the various mottled‐rind sources, three additional crosses were made between mottled lines. One was between DUD (ssp. *melo*, Duda'im Group) and PSR (ssp. *melo*, Inodorus Group; Figure 4g). Another was between QME (ssp. *collosus*) and PSR (Figure 4h), and the third between QME and PI414 (Figure 4i). In all three crosses, 100% of the F_2_ segregants (*n* = ~100) displayed mottled‐rind fruits, providing further supportive evidence that the various mottled rind patterns across melon diversity are allelic and conferred by the *MT‐2* gene.

Rind color and pigmentation pattern are primary components of melon fruit appearance. We previously reported *CmAPRR2* gene as a key regulator of rind and flesh pigment intensity in melon (Oren et al., 2019). That study benefitted from an integrative analysis of pigment variation across multi‐parental germplasm. Genomes resequencing of *MelonCore25* were used for a detailed analysis of haplotypic variation in the *CmAPRR2* gene and allowed detection of multiple independent predicted causative mutations across the diversity, supported by comprehensive allelism tests using the *HDA25* population. This high‐resolution multi‐parental analysis exposed the complex haplotypic variation in the *CmAPRR2* gene and resolved the unexpected lack of signal in genome‐wide association study (GWAS) for this conserved central pigment accumulation regulator (Oren et al., 2019). Co‐segregation analysis between the light‐rind phenotype and the ESL allele in the current study provides further support for the multi‐allelic model and predicted causality of an exon9 point mutation in *CmAPRR2* for the light rind of ESL (Figure 4d).

The mapping of the mottled rind trait was accomplished using a combination of segregating populations and a diverse collection (Figure 3e,f). The trait has been mapped to a ~115 kb interval with 14 annotated genes. These results coincide with previous mapping of the *MT‐2* gene by parallel analyses of different crosses, which supported the probable allelic nature of the mottled‐rind trait from diverse sources. Lv et al. (2018) mapped the ‘spotted’ rind trait in the Chinensis Group to 280 kb interval on chromosome 2. Pereira et al. (2018) described the mapping of mottled rind to the same genomic region in a cross between the non‐mottled rind ‘Védrantais’ (ssp. *melo*, Cantalupensis Group) and the mottled ‘Piel de Sapo’ (ssp. *melo*, Inodorus Group). Recently, Shen et al. (2021) mapped mottled rind to a 40.6 kb within the same interval harboring 6 genes, using a ssp*. agrestis* Chinensis Group accession as the trait donor. Conclusive evidence for the causative gene and sequence variants is yet to be provided. The mapping interval reported here overlaps with the 40.6 kb interval of Shen et al. (2021), and the detailed genomic variation available now for *MelonCore25* can assist in identifying the causative variation. We analyzed SNPs and SVs within the 6 candidate genes in the 40.6 kb interval (Tables S5 and S6). This region possesses 62 exonic SNPs, 21 of which are non‐synonymous polymorphisms, 14 are untranslated region (UTR) polymorphisms, and 27 are synonymous or splice‐site polymorphisms. Interestingly, none of these polymorphisms display a significant association with the mottled‐rind phenotype across the *MelonCore25* set. The broad multi‐parental analyses here and in previous studies support the notion that mottled‐rind variation is determined by a single gene across melon diversity, which could suggest a possible monophyletic origin of this trait. However, the lack of association of an exonic polymorphism across the 25 founders could also suggest that the variation is either regulated on other levels (such as expression or translation), or by multi‐allelic variation caused by different independent mutations in the causative gene, as previously described for the *CmAPRR2* gene (Oren et al., 2019). Resolution of this riddle could be achieved once the causative sequence variation is discovered and characterized.

#### Genetics of sugar accumulation

**Figure 5:**
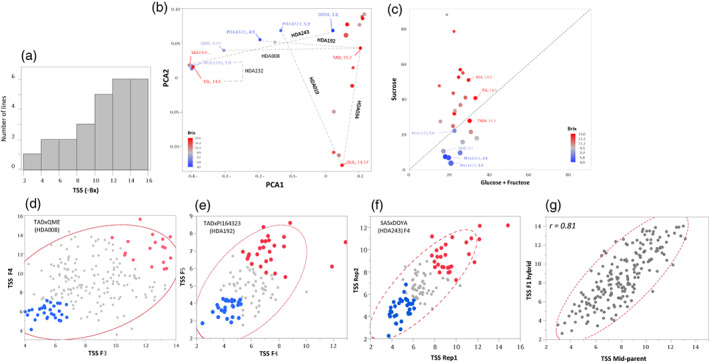
Variation in fruit sugars across *MelonCore25* and selected segregating populations for genetic mapping. (a) Distribution of total soluble solids (TSS) of mature fruits across *MelonCore25*. (b) Projection of accession TSS on the genetic PCA. Dashed lines represent crosses (and derived segregating populations) used for genetic dissection of fruit sugar accumulation. (c) Correlation between content of the monosaccharides, glucose and fructose, and disaccharide, sucrose, in mature fruits of *MelonCore25*. (d) Selection of tails segregants through the analysis of correlation between F_3_ and F_4_ lines of the cross TAD × QME (*HDA008*). High and low TSS tails are represented in red and blue, respectively. (e) Selection of tail segregants through the analysis of correlation between F_4_ and F_5_ lines of the cross TAD × PI164323 (*HDA192*). High and low TSS tails are represented in red and blue, respectively. (f) Selection of tail segregants through the analysis of correlation between replications in the F_4_ population from the cross SAS × DOYA (*HDA243*). High and low TSS tails are represented in red and blue, respectively. (g) Correlation between TSS of parental means (mid‐parent) and TSS of their F_1_ hybrids, across 190 F_1_s (*HDA20* population).

Fruit sweetness is the primary parameter defining melon quality and consumer preference. Much research has been devoted to obtaining improved understandings of carbohydrate metabolism and the complex genetic architecture of sugars accumulation and fruit sweetness (Argyris et al., 2017; Dai et al., 2011; Harel‐Beja et al., 2010; Leida et al., 2015; Schaffer et al., 1987). *MelonCore25* has been characterized for total soluble solids (TSS, °Bx) content of mature fruits grown in replicated open‐field experiments. The mean TSS of the 25 accessions varied from 4 to 15 (Figure 5a). This variation is similar to the range displayed across our broader *GWAS180* collection (Gur et al., 2017), and reflects the wide spectrum of fruit sweetness within *C. melo* and the relevance of studying the genetics and physiology of sugar transport and accumulation within this species. Projection of TSS values on the genetic PCA of *MelonCore25* (Figure 5b) shows that fruit sweetness is not independent of population structure, as most of the ssp. *melo* accessions are sweet, with the exception of non‐sweet lines from the Flexuosus and Adzhur Groups, but the ssp. *agrestis* accessions in our core set contain a more equal representation of high and low TSS. Based on this integrated view, efforts were focused on several crosses between high and low TSS accessions that represent different combinations of subspecies and cultivar‐groups. The rationale was to characterize the genetic architecture of sugar accumulation in multiple crosses that represent melon diversity, which could facilitate a comprehensive view of the genetic architecture of fruit sweetness. For example, *HDA232* is a cross between two closely related ssp. *agrestis* accessions differing in TSS: ESL (Makuwa Group, Bx = 14.6) and PI161375 (Chinensis Group, Bx = 7.0), and therefore such a cross potentially represents allelic variation in a small number of major sugar QTLs. On the other hand, *HDA243* is an inter‐subspecific combination between genetically distant accessions, the sweet ssp. *agrestis* SAS (Makuwa Group, Bx = 14.0) and the non‐sweet ssp. *melo* DOYA (Flexuosus Group, Bx = 3.8), and is therefore expected to reflect variation in a large number of sugar QTLs. Examples of three selected segregating populations that were advanced for mapping sugars QTLs are provided in Figure 5(d–f). The TSS correlation between F_3_ and F_4_ family means from the cross between TAD (ssp. *melo*, Inodorus Group, Bx = 15.2) and the non‐sweet QME (ssp. *collosus*, feral, Bx = 8.0) is presented in Figure 5(d), with tail segregants from 208 F_3_:F_4_ families highlighted for QTL‐Seq analysis. Figure 5(e) shows the TSS correlation between F_4_ and F_5_ family means from the cross between TAD and a different non‐sweet accession, PI164323 (subsp. *melo*, Adzhur Group, Bx = 4.9). Tail segregants from this population were also selected for mapping the main sugar QTLs in this cross. Figure 5(f) presents the variation in TSS and tail segregants selection in the F_5_ population derived from the inter‐subspecific cross between the sweet SAS and the non‐sweet DOYA.

To expand our view on the inheritance of sugar accumulation using the multi‐parental framework, we analyzed TSS in the *HDA20* sub‐population composed of 20 parents from *MelonCore25* alongside their 190 half‐diallel F_1_s. In order to gain broad insight on the mode of inheritance of fruit TSS, each of the 190 F_1_s was compared with its parents. Various modes of inheritance were observed across the broad *HDA20* set (Figure S3); however, the highly significant correlation (*r* = 0.81) between mid‐parent and F_1_ TSS (Figure 5g) across the 190 diverse hybrid groups is indicative of a mostly additive inheritance of TSS through melon diversity. Taken together with the complex genetic architecture of fruit TSS, these results imply that the use of multiple bi‐parental populations could be an effective path to genetically dissect this trait and develop marker‐assisted selection protocols to breed sweet melons.

In addition to TSS measurements, *MelonCore25* was also analyzed for sugar composition. As previously shown (Burger & Schaffer, 2007; Schaffer et al., 1987), the sugar content continuum in melon is explained by the variation in sucrose rather than in its hexoses building blocks—glucose and fructose (Figure 5c). This unidirectional variation, reflected through the bivariate view of the hexoses versus sucrose content across *MelonCore25* (Figure 5c) is another approach to express variation in sugar composition. Of the total sugars in melon fruit flesh, there is a highly variable proportion of sucrose, with the sweet‐fruited accessions containing 85% sucrose and the non‐sweet only 15%. This variation could prove to be an effective substrate for establishing genetic studies focused on sugars metabolism in melon fruit.

Although there is a clear distinction between sweet and non‐sweet melon cultivar‐groups, previous studies suggest that the genetics of sucrose metabolism and accumulation is complex and most likely controlled by multiple QTLs with small effects rather than variation in major genes (Diaz et al., 2011; Leida et al., 2015). The physiological and biochemical characterization of sugar metabolism performed thus far in melon has provided many candidate genes related to sugar metabolic pathways and transport (Dai et al., 2011), but causative genes explaining the phenotypic variation are yet to be discovered through effective genetic analyses. Argyris et al. (2017) analyzed multiple segregating populations from two crosses between sweet and non‐sweet parents, for mapping sugar‐accumulation QTLs. This approach is expanded here by targeting multiple crosses from the *HDA25F*_*2*_ *s* set. We selected several crosses between high‐ and low‐sugar parents from several inter‐subspecific and intra‐subspecific combinations (Figure 5b). Using the genome assemblies of all the participating parents in these crosses, parallel QTL‐Seq analyses can be performed using tail segregants (Figure 5d–f), and efficient annotations of sugar accumulation QTL intervals can be anticipated in these populations. In addition to the complete representation of SNP and short InDels variation in these populations, a detailed comparative analysis of SV within QTL intervals or presence–absence variation analysis of sugar metabolism and transport genes can be further analyzed and associated with phenotypic variation.

#### Genetics of resistance to soil‐borne pathogens

##### Variation in resistance to Fusarium wilt and haplotypic variation in Fom‐2 gene across MelonCore25

**Figure 6:**
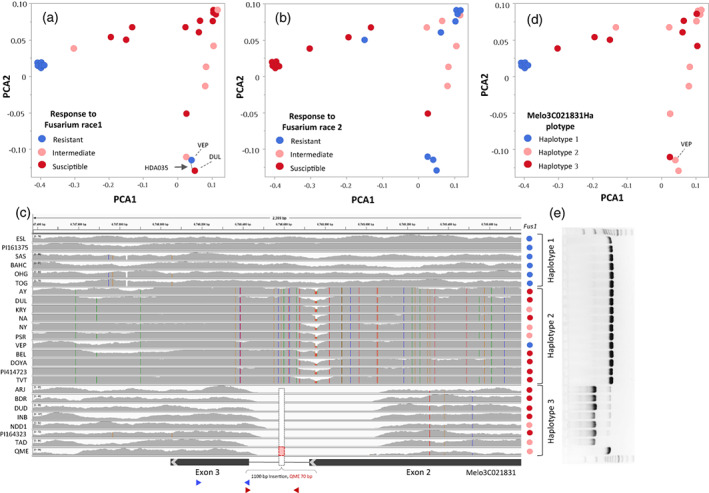
Variation in response to artificial inoculation with *Fusarium oxysporum* f. sp. *melonis* races 1 and 2 and haplotypic variation in the *FOM‐2* gene across *MelonCore25*. (a) Projection of accession response to inoculation with Fusarium race 1 on the genetic PCA. (b) Projection of accession response to inoculation with Fusarium race 2 on the genetic PCA. (c) Haplotypic variation in the *FOM‐2* gene (MELO3C021831) across *MelonCore25*. Vertical colored lines are SNPs. Gray histograms reflect short‐read depth. The dashed rectangle in haplotype 3 is the1100‐bp insertion discovered through *Nanopore* sequencing and *de novo* assemblies. Remarkably, a ~500‐bp deletion was initially indicated in this region based on absence of Illumina short‐read alignments. The actual insertion was discovered only based on the long‐read assemblies (Figure S5). Colored dots correspond to the Fusarium race 1 response as presented in (a). Part of the gene model is presented below the haplotypic view. Triangles below the gene model are PCR primers for FOM‐2 CAPs markers. Blue: marker from Oumouloud et al. (2015). Red: CAPs marker developed for the Newe Ya‘ar breeding program (Table S8). (d) Projection of accession *FOM‐2* haplotype on the genetic PCA. (e) Gel image of polymerase chain reaction (PCR) validation of the 1100‐bp InDel.

Fusarium wilt is an important disease affecting melon production, caused by the soil‐borne fungus *Fusarium oxysporum* f. sp. *melonis* (*F. o. melonis*). We characterized the response of young seedlings of the *MelonCore25* set to artificial inoculation with *F. o. melonis* races 1 and 2. For both pathogen races, a wide spectrum of responses from resistant to susceptible was observed and there was, as expected, a differential response to the two races (Figure 6a,b). Projection of the disease‐symptom indices on the genetic PCA displayed a division of response to race 1 by subspecies, such that the ssp. *agrestis* accessions were resistant and all but one of the ssp. *melo* accessions were intermediate or highly susceptible. The differential response to the races was most apparent among the *agrestis* accessions, that were resistant to race 1 and highly susceptible to race 2. A major gene (*Fom‐2*) conferring resistance to *F. o. melonis* races 0 and 1 has been mapped and positionally cloned (Joobeur et al., 2004). Genetic marker was developed within the *Fom‐2* gene for marker‐assisted selection in melon breeding (Wang et al., 2011). We used our multi‐parental platform to evaluate cases of limited predictive value of the *Fom‐2* marker observed across different breeding germplasm. The 25 genome assemblies allowed us to obtain a detailed alignment of the *Fom‐2* (Melo3C021831) gene sequence and detect multiple polymorphisms, including a large InDel between exon2 and exon3 (Figure 6c). In all, we found 45 polymorphic sites within exons of the *Fom‐2* gene, 26 of which were non‐synonymous polymorphisms resulting in different types of effects on the expected protein sequence (Table S7). Interestingly, this extensive sequence diversity is translated into only three main haplotypes across the *MelonCore25* set. Haplotype 1 is specific and common to the ssp. *agrestis* accessions in our core set and is similar to the DHL92 reference in this region (Figure 6c,d). Haplotype 2 is distributed across the different cultivar‐groups within ssp. *melo*, and characterized by multiple SNPs along the gene that are part of a linkage disequilibrium (LD) block. Haplotype 3 is common to ssp. *melo* lines from different cultivar‐groups and contains an insertion of ~1100 bp compared with the reference, and a few additional SNPs in the LD block (Figure 6c,d). While we found a significant association between these *Fom‐2* haplotypes and Fusarium race 1 resistance across *MelonCore25*, none of the polymorphisms independently explained the variation (Figure 6). The only exception to this haplotypic association is VEP (ssp. *melo*, Cantalupensis Group; Figure 6c: Haplotype 2, resistant to race 1), suggesting that additional genes are involved. The cross between VEP and the related susceptible DUL (ssp. *melo*, Reticulatus Group, Haplotype 2) is therefore an informed selection of a potential population for mapping these other genetic loci (Figure 6a).

##### Genetic analysis of resistance to *Fusarium oxysporum f. sp. radicis‐cucumerinum* (FORC)

**Figure 7:**
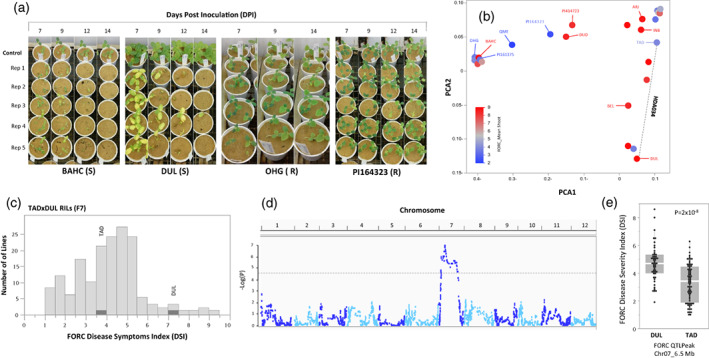
Variation in response to *Fusarium oxysporum* f. sp. *radicis‐cucumerinum* (FORC) across *MelonCore25* and QTL mapping of TAD × DUL RILs. (a) Experimental design and examples of disease response of susceptible (S) and resistant (R) accessions, 7–14 days post‐inoculation. (b) Projection of the responses of the 25 accessions to FORC on the genetic PCA. The dashed line marks a cross, HDA034, used for the quantitative trait loci (QTL) mapping. (c) Frequency distribution of FORC disease symptoms index (DSI) across 153 TAD × DUL RILs. Responses of parents are marked. (d) Manhattan plot for linkage mapping of response to FORC in the TAD × DUL RILs. (e) Allelic effect of the chromosome 7 QTL. For both respective genotypic groups, DUL and TAD, the two gray boxes represent the standard deviation and the blank spaces in‐between the allelic mean.

Fusarium root and stem rot caused by the fungus FORC is a disease of greenhouse‐grown cucumbers and melons (Punja & Parker, 2000; Vakalounakis et al., 2005). Genetic variation in the response to artificial inoculation with FORC has been described (Cohen et al., 2015; Elkabetz et al., 2016). Inoculation of FORC across the *MelonCore25* set revealed a wide spectrum ranging from susceptibility to resistance (Figure 7a). Projection of disease severity index (DSI) on the genetic PCA plot showed that the level of resistance to this pathogen is independent of population structure (Figure 7b). Multiple potential crosses between resistant and susceptible accessions are available in the *HDA25F*_*2*_ *s* set, and we used the RILs population developed from the cross between TAD (ssp. *melo*, Inodorous Group, intermediate resistance) and DUL (ssp. *melo*, Reticulatus Group, susceptible; Oren et al., 2019) to genetically dissect this trait. The RILs population displayed transgressive segregation in response to FORC inoculation, with lines more resistant than TAD or more susceptible than DUL (Figure 7c). This population was genotyped with 89 343 GBS SNPs defining 2853 recombination bins (Oren et al., 2019), and through whole‐genome QTL mapping analysis we identified a major QTL on chromosome 7 (Figure 7d) that explains 22% of the FORC DSI variation (*P* = 2 × 10^−8^; Figure 7e). We used the parental *de novo* assembled genomes and derived melon variome database to extract informative InDel markers for further backcrossing to validate and fine‐map this QTL.

##### Variation in response to charcoal rot (*Macrophomina phaseolina*) across *MelonCore25*

We recently completed a multi‐environment screen of *MelonCore25* for resistance to the soil‐borne fungal pathogen *Macrophomina phaseolina*, which causes a disease often referred to as charcoal rot (Cohen et al., 2022). The frequency distribution of cross‐environment average DSI of the 25 accessions is plotted in Figure S4(a). The whole spectrum from resistant to highly susceptible was observed across *MelonCore25*. Projection of the DSI values on the genetic PCA (Figure S4b) highlights the correlation between intra‐specific classification (ssp. *melo* and *agrestis*) and resistance level, such that most ssp. *agrestis* showed tolerance or moderate susceptibility, while most ssp. *melo* showed moderate or high susceptibility. Potential crosses are available between the tolerant and susceptible accessions in the *HDA25F*_*2*_ *s* set, and as such are a good substrate for genetic dissection of this trait.

### A unified framework for trait dissection in the pan‐genomic era

As many horticulturally important traits are polygenic, the availability and utilization of multi‐parental resources is required for comprehensive genetic dissection of natural variation and detection of a repertoire of favorable alleles for crop breeding. Melon genetic and genomic research has been much enhanced since the release of the first melon genome 10 years ago (Garcia‐Mas et al., 2012), followed by extensive re‐sequencing of hundreds of melon accessions (Dafna et al., 2021; Liu, Gao, et al., 2020; Zhao et al., 2019). Five additional high‐quality melon genome assemblies that can be used as references for this species were published over the past 4 years (Ling et al., 2021; Pichot et al., 2022; Yang et al., 2020; Yano et al., 2020; Zhang et al., 2019). However, the recent expansion of the pan‐genome concept, based on the realization that multiple genomes capturing the diversity within species are required for complete representation of the genomic variation (Bayer et al., 2020; Coletta et al., 2021; Ho et al., 2020; Li et al., 2022), suggest that the SV layer is not fully exposed in melon. We, therefore, developed and describe here a multi‐parental trait dissection framework, anchored to a novel pan‐genome constructed based on high‐quality assemblies of 25 genomes, representing melon diversity. Pan‐genomes have been described for several crop plants, highlighting the commonality and magnitude of within‐species SV (Gao et al., 2019; Jayakodi et al., 2020; Li et al., 2022; Liu, Du, et al., 2020; Qin et al., 2021; Song et al., 2020). The unique aspect of the current project is the creation of a unified common framework that integrates genetic diversity, phenotypic variation and genomic information. In addition to the traits described here, the *MelonCore25* set was phenotyped to fruit morphology traits (size and shape), rind and flesh color and pigment content (Gur et al., 2017), rind netting (Oren et al., 2020), fruit firmness, ripening behavior (Oren et al., 2022), and over 80 000 metabolomic and elemental features extracted from rind and flesh (Moing et al., 2020). Alongside the 25 genome assemblies and segregating populations covering all possible parental combinations, the genetic basis of these traits (and others) can now be studied more effectively. An important additional layer that can supplement the current framework is a comprehensive expression profiling (transcriptomes) of the *MelonCore25* accessions across different organs, tissues and developmental stages, which could provide an important layer for candidate‐gene discovery and improve genome annotations.

### Conclusions

We describe here a new resource for genomic research in melon. The pan‐genome constructed from 25 diverse accessions selected to represent the broad variation across *C. melo* is useful for comparative genomic analyses to address the evolution of the melon genome and the impact of breeding history. The integral connection of the pan‐genome with a set of crosses and segregating populations (*HDA25F*_*2*_, *n* = 300) is expanding the usability of this resource. For example, correlation between SV and parental crossability or fertility of F_1_s can be studied. Detailed analysis of relations between large genomic rearrangements and recombination frequency can be directly addressed using the SVs database and F_2_ populations. We demonstrate the primary potential of this integrated framework: genetic and genomic dissection of traits. The genomic information could also be useful for informed design of genome editing and trait introgression in breeding programs, taking into consideration the impact of SVs.

## EXPERIMENTAL PROCEDURES

### Plant materials and field trials

#### MelonCore25

This research is centered on the core set of 25 diverse melon accessions (Figure 1; Table 1) that were selected based on a comprehensive genotypic and phenotypic characterization of our broader *GWAS180* panel (Gur et al., 2017). The accessions for this core set were selected based on the multiple genotypic and phenotypic criteria previously described (Dafna et al., 2021; Gur et al., 2017). Briefly, an initial tentative set (*n* = 40) was constructed to represent all the cultivar‐groups of melon (Burger et al., 2009; Pitrat et al., 2000). Phenotypic profiles were then used as the second primary factor; the preliminary core set was projected on the distribution of the different traits to ensure a phenotypic spectrum that is well‐captured in the core panel (as illustrated by Gur et al., 2017). Following required adjustments and narrowing of the set to *n* = 30, based on the first two steps, the final set was selected to meet the 25‐accession target, taking into account maximum polymorphism information content value and uniform distribution on genetic diversity plots. Genetic PCA coordinates of the 25 founders are available in Table S1.

#### HDA25

The creation of the diverse, 25‐way half‐diallel population, resulting in 300 F_1_ hybrids that represent all possible parental combinations, was previously described by Dafna et al. (2021).

#### HDA20

A subset of *HDA25*, *HDA20* is composed of 20 representative lines of the 25 members of *HDA25*. The 20‐way half‐diallel population of *HDA20* resulted in 190 F_1_s. This subset was described by Dafna et al. (2021).

#### HDA25F_2_s

All 300 *HDA25* F_1_s were grown in a greenhouse at Newe Ya‘ar and subjected to self‐pollinations to produce 300 F_2_ populations.

#### PI414723 × Dulce RILs population (*HDA019*, F_7_)

A bi‐parental population of 99 RILs from a cross between the mottled‐rind PI414723 (ssp. *agrestis*, Momordica Group) and the non‐mottled‐rind ‘Dulce’ (ssp. *melo*, Reticulatus Group) was previously described (Harel‐Beja et al., 2010).

#### Tam Dew × Dulce RILs population (*HDA034*, F_7_)

A bi‐parental RILs population of 164 RILs from a cross between ‘Tam Dew’ (TAD; ssp. *melo* Inodorus Group) and ‘Dulce’ as described by Oren et al. (2020).

#### Tam Dew × Qishu Meshullash (*HDA008*, F_6_)

Tam Dew was crossed with Qishu Meshullash (QME, ssp. *Colossus*), in a greenhouse at Newe Ya‘ar and ~200 lines were advanced to the F_6_ generation by the single‐seed‐descent selfing scheme. Qishu Meshullash was collected from a feral population growing in Israel and was obtained from the Israel Gene Bank. It was identified as subsp. *collosus* based on the recent clarifications of this taxon by John et al. (2013) and Endl et al. (2018).

#### Tam Dew × PI164323 (*HDA192*, F_5_)

Progenies of the cross between the sweet‐fleshed Tam Dew and the non‐sweet accession PI164323 (ssp. *melo*, Adzhur Group) were grown in a greenhouse at Newe Ya‘ar and ~150 lines were advanced to the F_5_ generation by single‐seed‐descent selfing.

#### Sakata's Sweet × Doya's Faqqous (*HDA243*, F_5_)

The cross between the sweet‐fleshed ‘Sakata's Sweet’ (SAS, ssp. *agrestis*, Makuwa Group) and the locally grown, non‐sweet accession that we named ‘Doya's Faqqous’ (ssp. *melo*, Flexuosus Group) was grown in a greenhouse at Newe Ya‘ar and advanced to the F_5_ generation by single‐seed‐descent selfing.

#### PI161375 × Early Silver Line (*HDA232*, F_2_)

The cross between the mottled‐rind PI161375 (ssp. *agrestis*, Chinensis Group) and the non‐mottled‐rind Early Silver Line (ESL, ssp. *agrestis*, Makuwa Group) was grown in a greenhouse at Newe Ya‘ar and F_2_ seeds were produced as part of the *HDA25F*_*2*_ seed production.

All the above populations were grown in the open field or in the greenhouse at Newe Ya‘ar under common practices, as previously described (Gur et al., 2017; Oren et al., 2020).

### Trait evaluation

#### Sugars analysis

Five mature fruits per plot were sampled in multiple harvests, with a total of 10–15 fruits per accession being analyzed. Concentrations of TSS (expressed in °Bx) were measured on flesh samples from each of the five fruits separately, using a hand‐held refractometer (Atago, Tokyo, Japan). Approximately 1 g of frozen mesocarp tissue, taken from the center‐equatorial portion of the fruit, was placed in 80% EtOH, and soluble sugars (glucose, fructose and sucrose) were extracted and analyzed by high‐performance liquid chromatography as previously described (Petreikov et al., 2006).

#### Characterization of rind color and mottling pattern

Rind color intensity (light versus dark) and mottling (mottled versus smooth) were scored visually on young fruits on the vines at ~10–20 days post‐anthesis, and after harvest of mature fruit.

#### Response to fusarium wilt

Protocols for pathogen and plant inoculation were described in details by Burger et al. (2003). *Fusarium oxysporum* f. sp*. melonis* used for the inoculation was maintained on yeast extract medium at 27°C in the dark. Conidial suspensions for seedling inoculation were prepared by macerating 1‐week‐old cultures with 100 ml water (Cohen et al., 1996). Seeds of the *MelonCore25* accessions were sown in sandy soil. Two days after emergence, the seedlings were removed from the soil and washed under running tap water. The roots were pruned to approximately half their length, and the seedlings were then inoculated by dipping the roots in a conidial suspension (10^6^ conidia ml^−1^) for 2 min. The inoculated seedlings were then transplanted into 250‐ml pots containing new, disease‐free, sandy soil. Each accession was represented by 35 seedlings (five pots × seven plants per pot). The first wilting symptoms were evident 7 days after inoculation. The number of wilted plants was recorded twice a week for 2 weeks and disease incidence was calculated.

#### FORC inoculation and symptoms scoring

Pathogen and plant inoculation were described in detail by Cohen et al. (2015). Briefly, melon seeds were sown in autoclaved sandy soil. Four days post‐emergence, seedlings were removed from the soil and washed under running tap water. The roots were gently pruned and the seedlings were inoculated by dipping the roots in a suspension of 10^6^ conidia ml^−1^ for 5 min. The inoculated seedlings were then transplanted into 250‐ml pots containing clean sand and maintained in a growth room at a photoperiod regime of 12 h light/darkness. The seedlings were observed for 18 days following inoculation with FORC. The first disease symptom on ‘Dulce’ plants, leaf chlorosis, appeared 6 days after inoculation. Subsequently, the susceptible plants exhibited necrosis of the leaf margins, browning of the base of the stem, and wilting of the leaves and apical portion of the stem. The plant crown then became necrotic and the plant died. Eighteen days after inoculation, each plant was scored as symptomless (resistant), or as dead, wilted, necrotic or chlorotic (susceptible).

#### *Macrophomina phaseolina* inoculation and symptom scoring

Pathogen and plant inoculation were described in details by Cohen et al. (2022). Briefly, *M. phaseolina* (isolate no. NY 198) was isolated from melon plants. This isolate was tested routinely and is highly pathogenic. Melon plants at the age of 5 true leaves were inoculated, approximately 1 cm above ground level, by stabbing them with wooden toothpicks infested with *M. phaseolina* leaving the toothpick in the stab wound (Cohen et al., 2016). Disease severity was scored based on lesion development and size on a 0‐to‐5 scale, with 0 = symptomless plant, 1 = initial small (< 1 cm) lesion, 2 = 1–3 cm lesion, 3 = stem rotting of 2–4 cm, 4 = stem rotting of > 5 cm, 5 = plant dead, completely necrotic. Accessions having average scores of ≤ 2.0 were considered to be resistant to *M. phaseolina*, whilst those having average scores of ≥ 3.0 were considered to be susceptible.

### Statistical analyses

The JMP ver. 14.0.0 statistical package (SAS Institute, Cary, NC, USA) was used for all the general statistical analyses (i.e. frequency distributions, correlations, analyses of variance and mean comparisons).

### GWA mapping and QTL analysis

The GWA analysis of the mottled rind trait on the *GWAS180* panel was performed in TASSEL v.5.2.43 (Bradbury et al., 2007) using the mixed‐linear model function, as previously described by Gur et al. (2017). Linkage analysis and QTL mapping were performed using R/qtl (Broman et al., 2003) as described in Oren et al. (2020).

### DNA preparation and long‐read Nanopore sequencing

High‐molecular‐weight (HMW) DNA extraction and size selection were carried out either using a modified CTAB protocol as previously described in Oren et al. (2022) or using Macherey‐Nagel NucleoBond HMW DNA kit (REF 740160, www.mn‐net.com). Library preparation was carried out following ONT guidelines as described in Oren et al. (2022) and sequenced on MinION FLO‐MIN106D flow cells. Base calling was done using the GPU version of Guppy (V5.0.11, Oxford Nanopore Technologies, Abingdon, UK) using the HAC model.

### Genome assembly and scaffolding

The ONT reads were assembled using the Flye assembler v2.9 (Kolmogorov et al., 2019), by a previously detailed method (Oren et al., 2022), with the following adjustments: following initial contig assembly, only one round of ONT‐based polishing was carried out using the built‐in Flye polishing function, followed by one round of Illumina reads polishing using Pilon v1.24 (Walker et al., 2014). Reference‐guided scaffolding of contigs into pseudomolecules was carried out using RagTag v2.1.0 (Alonge et al., 2019) with the ‘Harukei‐3’ melon as a reference (Yano et al., 2020). We used the ‘Harukei‐3’genome to avoid possible inverted assembly regions on chromosome 6 of DHL92 (Yang et al., 2020).

### Characterization of SVs across *MelonCore25* and 300 HDA25 crosses

The SVs were characterized by whole‐genome alignment of each of the *MelonCore25* genomes to the DHL92 v4.0 genome using nucmer4 (Marçais et al., 2018) with –maxmatch –l 100 –c 500 parameters. The resulting delta file was then analyzed for InDels with command‐line scripts of Assemblytics (v1.2.1) (Nattestad & Schatz, 2016), setting unique sequence length for anchors at 10 kb. For short InDels database, InDel size was limited to a range of 15–2000 bp—fragment sizes that can be effectively amplified using standard PCR assay. The Assemblytics output tables of all *MelonCore25* lines were concatenated, filtered to InDel‐type variants and split by accession to create a matrix of 194K InDels with a separate presence/absence column per accession for each InDel. A simple conditional script was used to list the presence/absence of each InDel across the 300 diallel hybrids obtained from *MelonCore25*. The final InDel table was composed of 115 802 InDels, their attributes and 325 presence/absence columns (25 accessions and 300 hybrids; Table S3).

### Validation of selected InDels and genotyping of *MT‐2* and Fom‐2 by PCR

Twenty‐two InDels ranging in size from 16 to 1209 bp that showed polymorphism between DUL and PI414 were selected from our SV database (Table S4), and generated using Assemblytics (v1.2.1) (Nattestad & Schatz, 2016) for validation. Primers were designed using Gene Runner version 6.5. PCR, with an annealing temperature of 56°C, was performed on genomic DNA of Dulce, PI414 and their F_1_, using 2xPCRBIO HS Taq Mix Red (PCRBIOSYSTEMS, London, UK). The products were separated on a 2.5% or 1.2% agarose gel for 1–2 h (Figure S2; Table S4). Primers for *MT‐2* and *FOM‐2* markers are listed in Table S8.

### Gene models annotation

Draft gene models were annotated using a lift‐over approach from two reference sources, the ssp. *melo* Reticulatus line ‘Harukei‐3’ (Yano et al., 2020) and the ssp. *agrestis* line HS (Yang et al., 2020) to capture genes from both subspecies. Liftoff v1.6.3 (Shumate & Salzberg, 2021) was run separately for each reference with lenient thresholds on gene coverage and identity (−exclude_partial ‐a 0.5 ‐s 0.5 ‐copies), to prevent stringent filtering before determining values that accurately capture the pan and core genes repertoire. Custom filtration is possible on the annotation files based on sequence similarity values in the attributes column of the gff3 file (coverage; extra_copy_number;sequence_ID). To keep unique gene models from each gff3 file, the gene levels were converted to a bed file using gfftobed (v1.3, https://github.com/jacobbierstedt/gfftobed) and BEDTools intersect was used to mark overlapping gene models lifted over from ‘HS’ and ‘Harukei‐3’ (Quinlan & Hall, 2010). In the overlapping areas, gene models from the ‘HS’ were removed as the annotation methods used for the ‘Harukei‐3’ assembly were more rigorous. Prior to merging unique annotations from both sources, an ‘HS’ prefix was added to all ‘HS’ gene models, followed by sorting and tidying. Filtering, naming and sorting of the gene models was done with AGAT toolkit (Version v0.8.0, https://www.doi.org/10.5281/zenodo.3552717) and tidying was done with genometools gff3 tool (v.1.6.2, https://github.com/genometools/genometools).

## AUTHOR CONTRIBUTIONS

AG conceived the research plan; EO, AD and AG designed the experiments; US, SO, AD, JB and AG were responsible for development of plant genetic materials; AD, EO, GT, TI, AM, IH and AG performed the field experiments and collected the data; AD was responsible for digital data collection; ME, SO and RC performed phytopathology screens; YT and AAS contributed to sugars analyses; GT performed DNA markers analyses. EO, CR, TL and ESB planned and performed the genomic sequencing and *de novo* assembly pipeline. EO analyzed the genomic data and assembled the genomes. EO and AG analyzed the results. AG and EO wrote the manuscript. All authors discussed the results, reviewed and approved the final version of the manuscript.

## CONFLICT OF INTEREST

The authors declare no conflict of interest.

## Supporting information


**Figure S1.** Workflow for using the pan‐genome and multi‐parental mapping framework.
**Figure S2.** PCR validation of 22 InDels. InDel numbers correspond to those in Table S4.
**Figure S3.** Mode of inheritance of TSS across the *HDA20* set: 190 hybrids and their parents.
**Figure S4.** Variation in disease severity index (DSI) to *Macrophomina phaseolina* across *MelonCore25*. (a) Frequency distribution of DSI across *MelonCore25*. (b) Projection of DSI on the genetic PCA. Diverse crosses are indicated with dashed lines and hybrid code (HDA number).
**Figure S5.** Comparisons between ONT and Ilumina reads alignments across 8 accessions carrying the FOM 2 insertion. Purple boxes within ONT reads represent the ~1100 bp insertions.

**Table S1.** Nanopore long‐read sequencing statistics and genetic PCA coordinates of MelonCore25

**Table S2.** Large SVs across *MelonCore25*

**Table S3.** Short InDels across *MelonCore25* and 300 *HDA25* crosses

**Table S4.** List of 22 InDels validated by PCR

**Table S5.** SNPs and short exonic polymorphisms within genes in the mottled‐rind interval (*MT‐2*) on chromosome 2, across *MelonCore25*

**Table S6.** SVs within the mottled‐rind interval (*MT‐2*) on chromosome 2, across *MelonCore25*

**Table S7.** Exonic polymorphisms in the MELO3C021831 *FOM‐2* gene, across *MelonCore25*

**Table S8.** Primers for the *MT‐2* and *FOM‐2* markers

## Data Availability

All the data supporting the findings of this study are available within the paper and within its supplementary materials published online. Raw sequences and FASTA files of genome assemblies can be found at NCBI BioProject PRJNA726743. Gene annotation files for each of the genomes (gff format) are available at Zenodo repository https://doi.org/10.5281/zenodo.7212598.
