# Supplementary material for: Pan‐genome and multi‐parental framework for high‐resolution trait dissection in melon (Cucumis melo)
Source: Plant J. 2022 Nov 23;112(6):1525–42. doi: 10.1111/tpj.16021 (PMC10100132; doi:10.1111/tpj.16021)

Sup. Figure 1: Workflow for using the pan-genome and multi-parental mapping framework

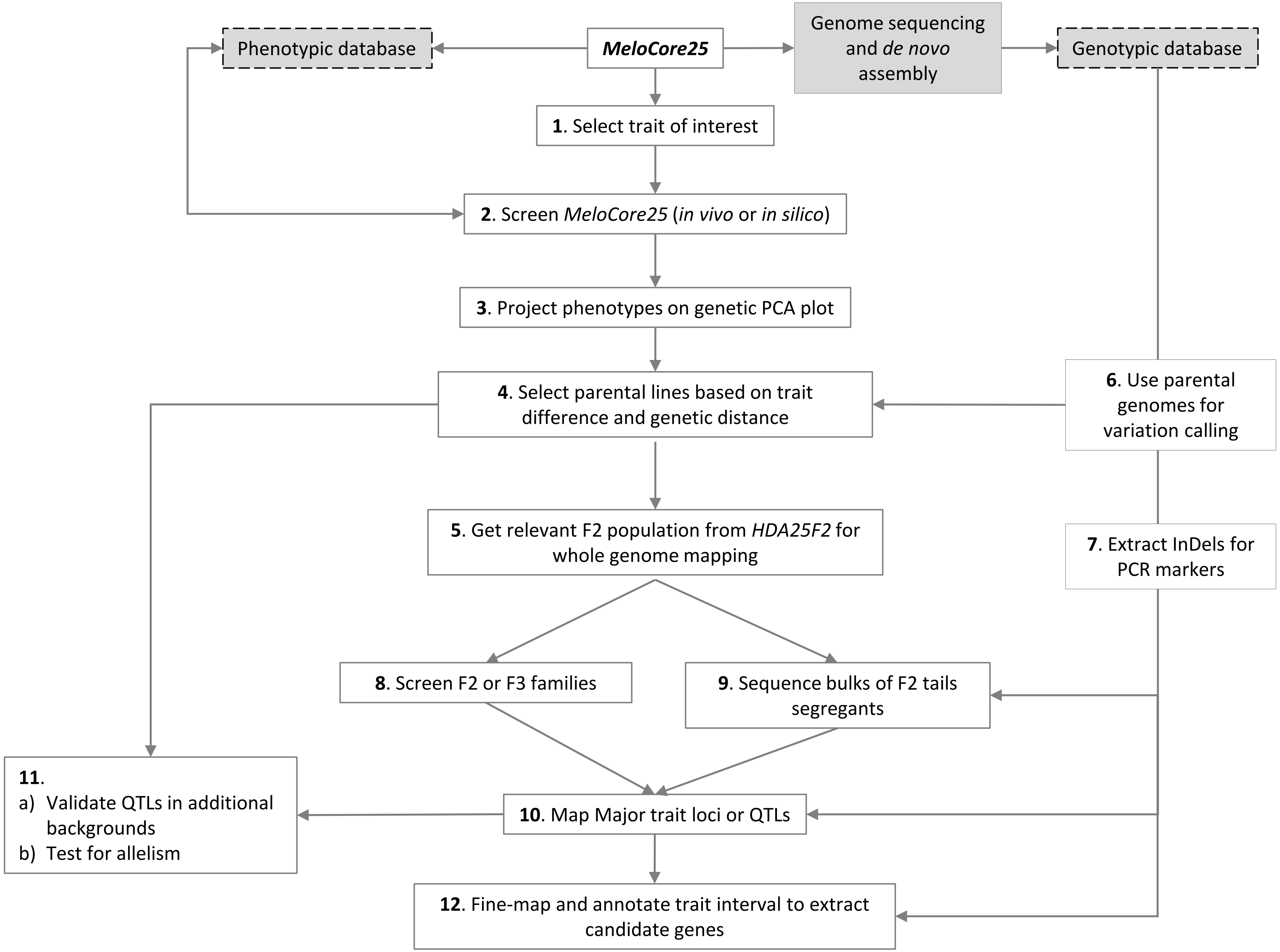

Sup. Figure 2: PCR validation of 22 Insertion-Deletion (InDels) identified in the pan-genome

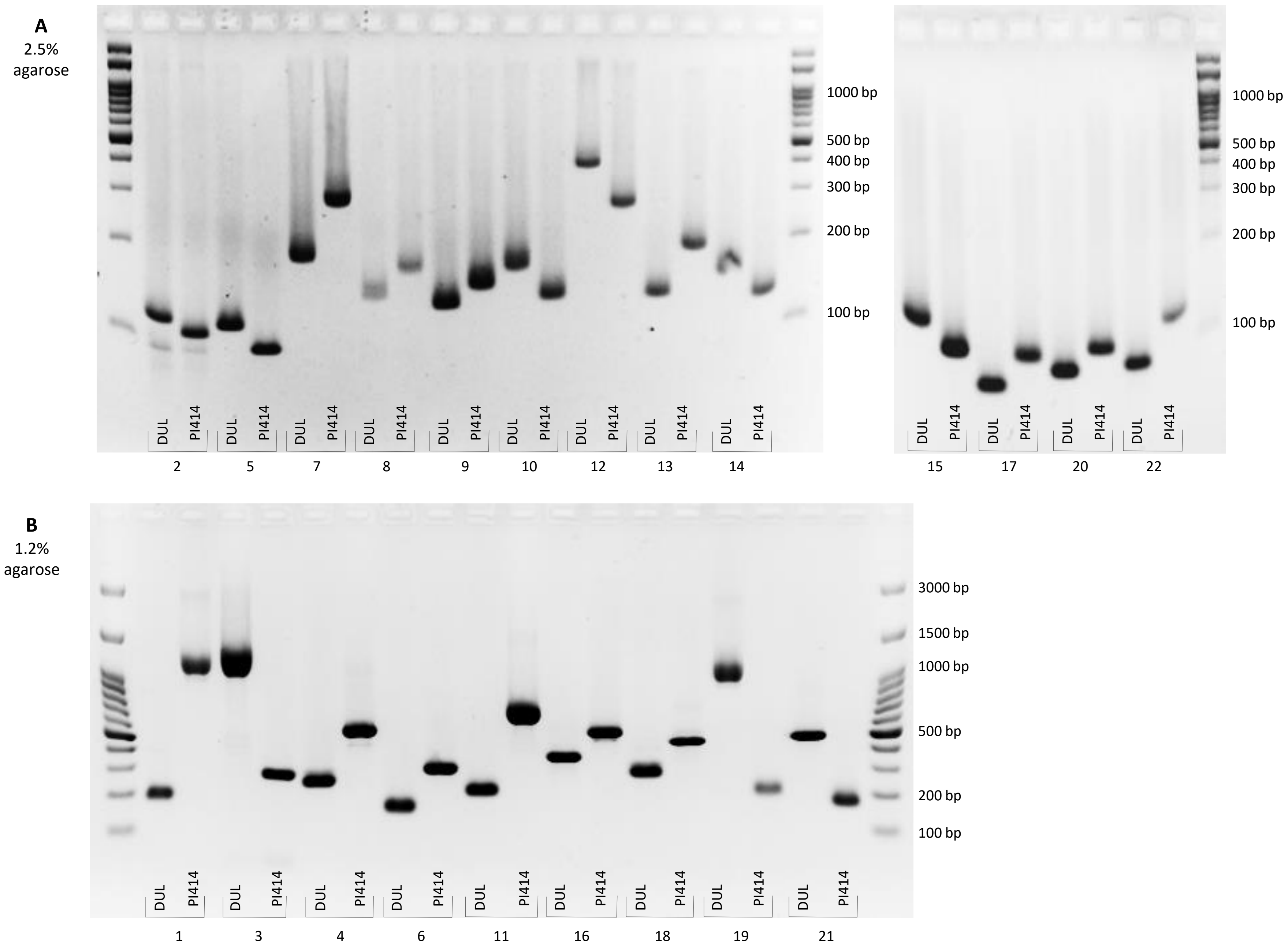

Sup. Figure 3: Inheritance of TSS across *HDA20* set: 190 hybrids and their parental lines. MP-Mid-Parent (parental Mean TSS value). LowP-Low Parent. HighP- High Parent

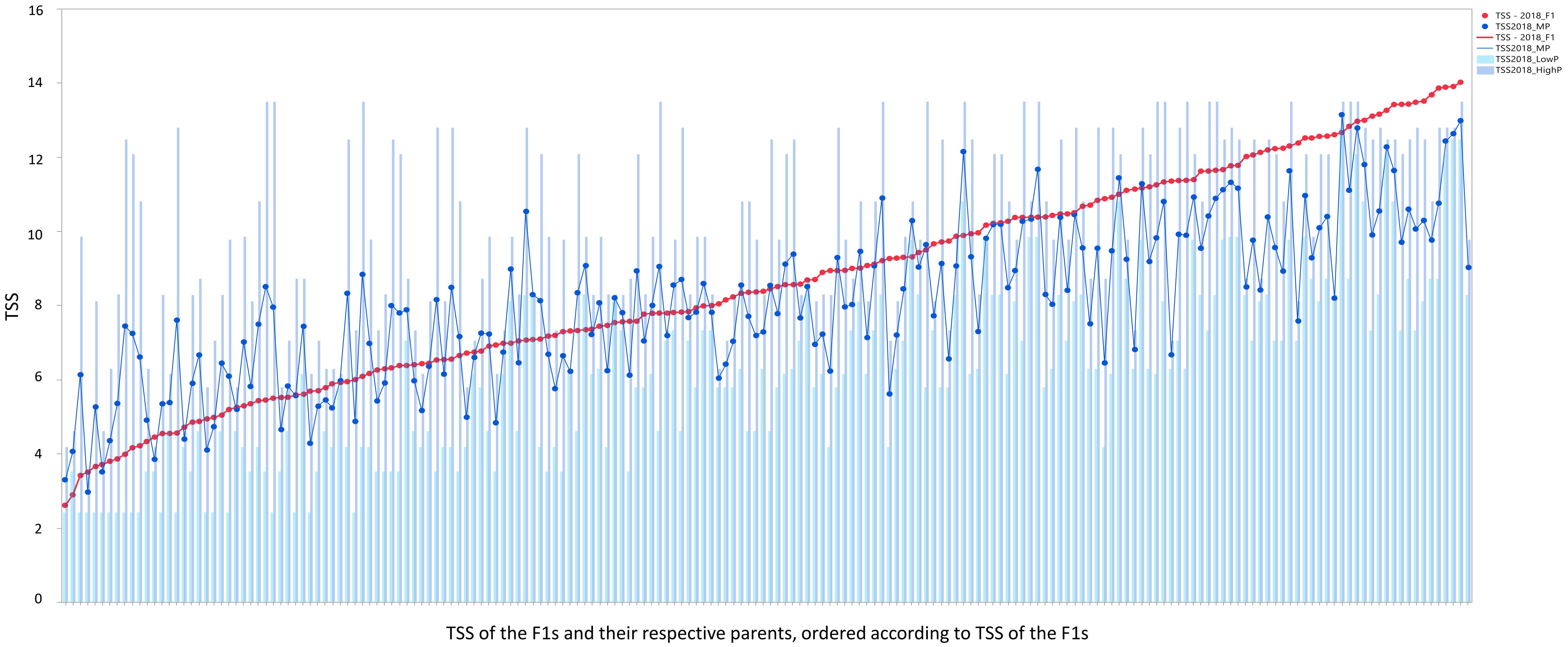

Sup. Figure 4: *Macrophomina* Disease symptoms Index (DSI) across *MelonCore25*

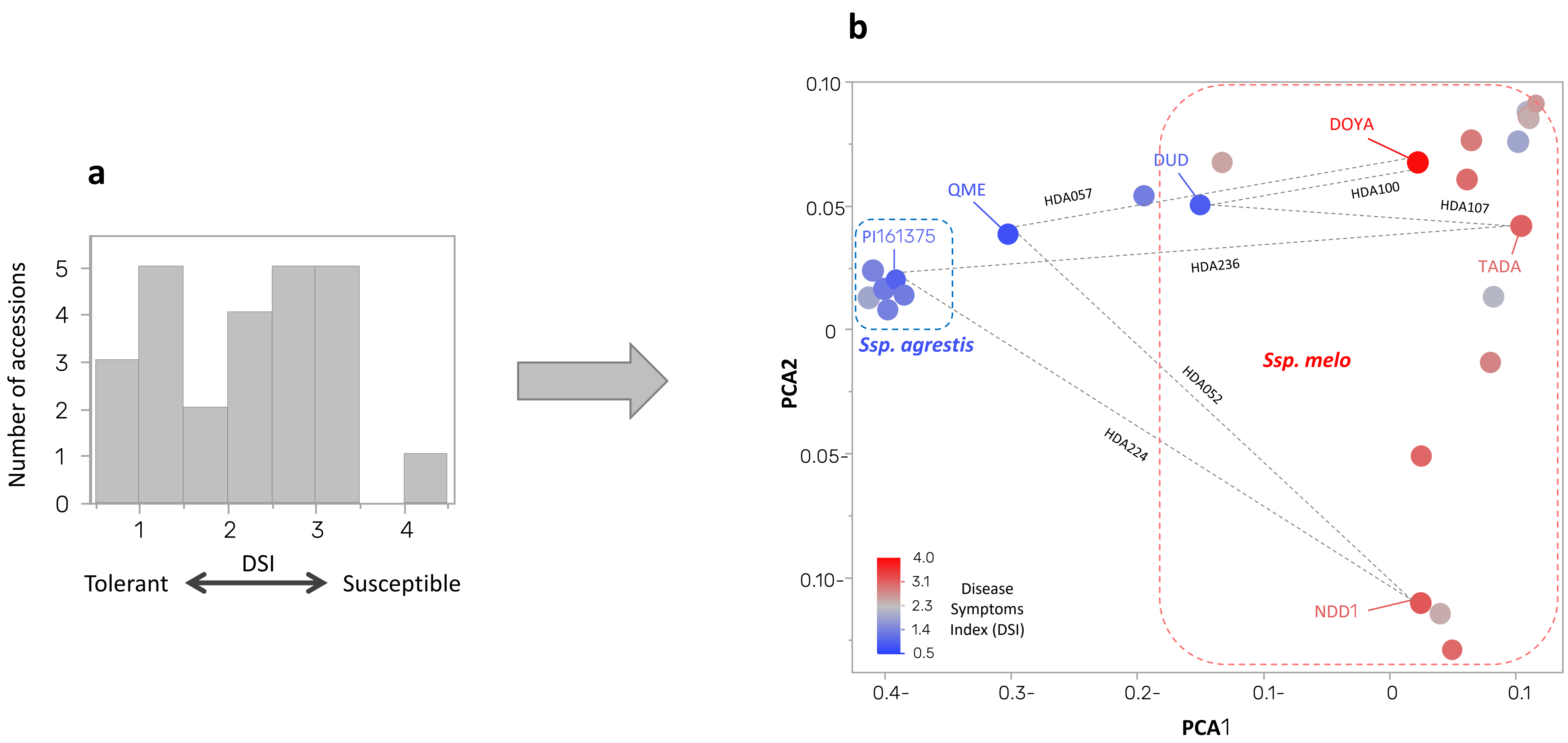

Sup. Figure 5: Comparisons between ONT and Ilumina reads alignments across 8 accessions carrying the *FOM-2* insertion.

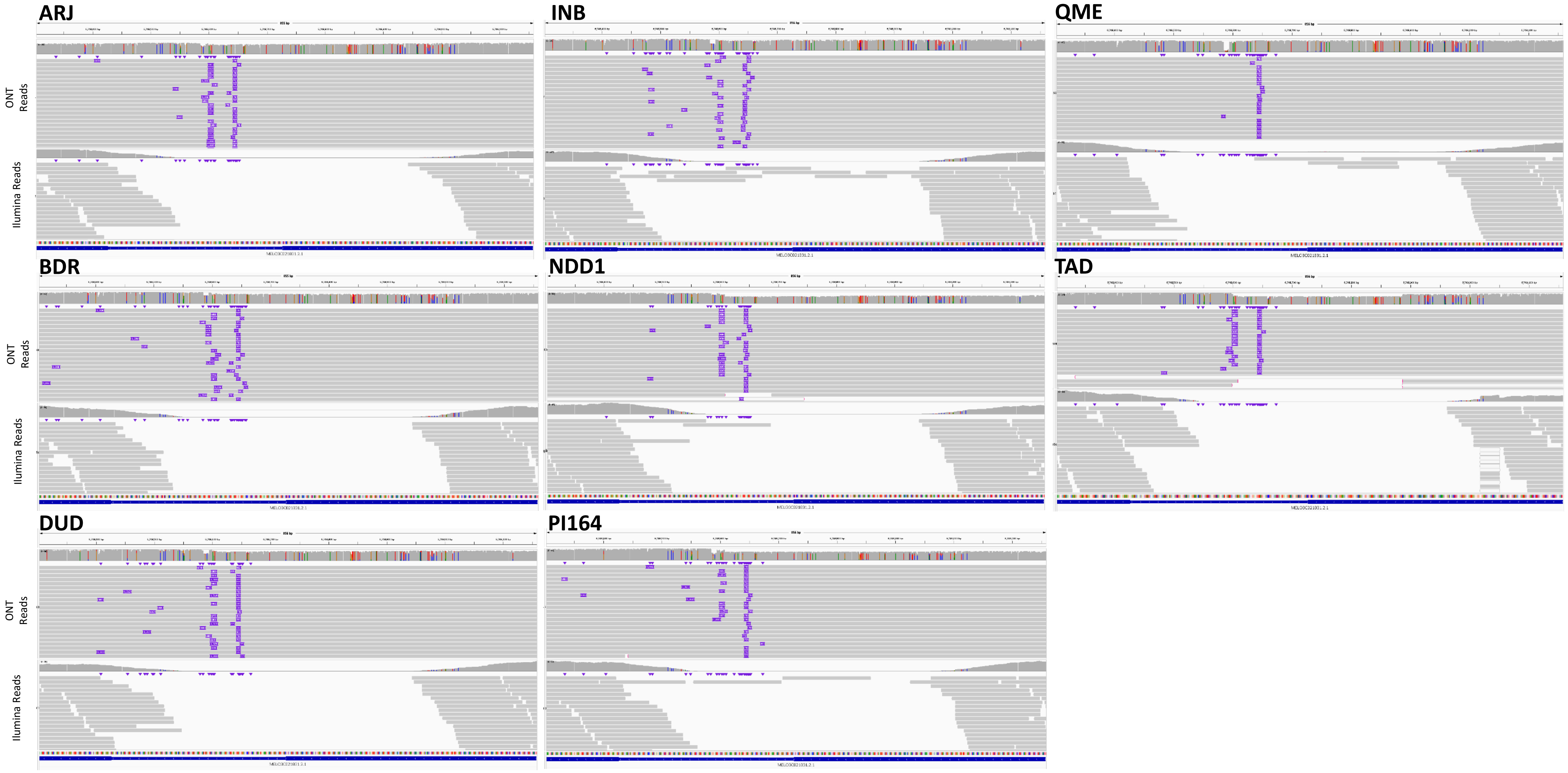

Supplement: Supplementary file 1 — Figure S1. Workflow for using the pan‐genome and multi‐parental mapping framework. Figure S2. PCR validation of 22 InDels. InDel numbers correspond to those in Table S4. Figure S3. Mode of inheritance of TSS across the HDA20 set: 190 hybrids and their parents. Figure S4. Variation in disease severity index (DSI) to Macrophomina phaseolina across MelonCore25. (a) Frequency distribution of DSI across MelonCore25. (b) Projection of DSI on the genetic PCA. Diverse crosses are indicated with dashed lines and hybrid code (HDA number). Figure S5. Comparisons between ONT and Ilumina reads alignments across 8 accessions carrying the FOM 2 insertion. Purple boxes within ONT reads represent the ~1100 bp insertions. [file TPJ-112-1525-s005.pdf]
